# A Constitutive Model of Time-Dependent Deformation Behavior for Sandstone

**DOI:** 10.3390/ma16010135

**Published:** 2022-12-23

**Authors:** Chongfeng Chen

**Affiliations:** 1 Xi’an Research Institute, China Coal Technology and Engineering Group Corp, Xi’an 710077, China; neuccf@163.com; 2 School of Civil and Transportation Engineering, Henan University of Urban Construction, Pingdingshan 467041, China; 3 School of Resources and Civil Engineering, Northeastern University, Shenyang 110004, China

**Keywords:** time-dependent deformation of sandstone, crack propagation, macroscopic fracture, microscopic heterogeneity, viscoelastic plastic deformation, damage evolution

## Abstract

Considering sandstone’s heterogeneity in the mesoscale and homogeneity in the macroscale, it is very difficult to describe its time-dependent behavior under stress. The mesoscale heterogeneity can affect the initiation and propagation of cracks. Clusters of cracks have a strong influence on the formation of macroscale fractures. In order to investigate the influence of crack evolution on the formation of fractures during creep deformation, a time-dependent damage model is introduced in this paper. First, the instantaneous elastoplastic damage model of sandstone was built based on the elastoplastic theory of rock and the micro-heterogeneous characteristics of sandstone. A viscoelastic plastic creep damage model was established by combining the Nishihara model and the elastoplastic damage constitutive model. The proposed models have been validated by the results of corresponding analytical solutions. To help back up the model, some conventional constant strain rate tests and multi-step creep tests were carried out to analyze the time-dependent behavior of sandstone. The results show that the proposed damage model can not only reflect the time-dependent viscoelastic deformation characteristics of sandstone, but also provide a good fit to the viscoelastic plastic deformation characteristics of sandstone’s creep behavior. The damage model can also reproduce the propagation process of mesoscopic cracks in sandstone upon the damage and failure of micro-units. This research can provide an effective tool for studying the propagation of microscopic cracks in sandstone.

## 1. Introduction

Many studies have demonstrated that quartz-rich rock can deform and eventually fail under a constant differential stress (*σ*_1_–*σ*_3_) over extended periods of time, a phenomenon known as brittle creep (or time-dependent deformation, abbreviated to TDD in the following) [1], especially for sandstone (a rock often seen in the roofs and floors of coal seams) [2,3]. Sandstone taken from deep coal mines and deformed in creep experiments generally exhibits three regimes of strain vs. time: (1) primary creep, or decreasing strain rate stage; (2) secondary creep, or constant strain rate stage; (3) tertiary creep, or increasing strain rate stage ending in failure [4,5]. However, some researchers have divided the creep behavior of rock into three sections: elastic instantaneous deformation, viscoelastic TDD, and viscoplastic TDD [6,7,8,9,10,11]. This categorization—identifying three kinds of deformation—is convenient for building a constitutive model that can be used to reproduce the creep deformation of rock and further understand the failure evolution of rock.

There has been considerable research on the viscoelastic plastic deformation of rock. Shao et al. [12,13] proposed a damage model to describe the elastic-plastic short term behavior and the elastoplastic time-dependent behavior. Their damage law is described in the framework of irreversible thermodynamics. The proposed constitutive relationship could be very suitable for semi-brittle rock material with the help of non-associated plastic flow. Zhao et al. [11,14] have separated the total creep strain into instantaneous elastic strain, instantaneous plastic strain, time-dependent viscoelastic strain and time-dependent visco-plastic strain based on experimental data. Additionally, Prof. Zhao proposed a nonlinear elastoviscous plastic creep model on the basis of element models. The results reveal that the strain curves from the creep model agree well with experimental results and give a precise description of the three stages of creep. Additionally, numerous papers have been published on the description of full stages of rock creep behavior [10,15,16,17,18,19,20,21,22,23,24,25]. However, most of the published papers focus mainly on strain-time curve fitting or seek to reproduce the full stages of creep behavior. Even though all of them have achieved good agreement with experimental results, we still have no idea how crack evolution influences the formation of fractures in the rock. As a result, some researchers have studied crack evolution before rock failure, going beyond matching the numerical and laboratory strain curves. Xu et al. [26,27] adopted a Norton–Bailey equation to construct the creep constitutive model, which was able to characterize the time-dependent creep deformation quite well. The creep model could also illustrate the failure of rock mass slope using the Norton–Bailey creep equation and the time-independent damage evolution law. In addition, Fu et al. [28] have constructed a three-dimensional discrete element grain-based model to simulate the fracture evolution of rock around underground excavations. The simulation results agree well with those found in the laboratory. Wang et al. [25,29] built a grain-based stress corrosion model based on the distinct element method to study time-dependent failure during creep tests for brittle rock. The crack propagation and the damage evolution induced by stress concentration before the creep failure are discussed in these papers. In addition, some other researchers have worked on the creep behavior of engineering structures such as tunnels and roadways [30,31,32].

Although, there are some published articles related to the macroscopic failure behavior or viscoelastic plastic properties of rock induced by microscopic damage evolution during time-independent deformation [33,34,35,36,37,38,39,40,41], few studies closely combined the microscopic damage evolution of rock with macroscopic TDD and viscoelastic plastic mechanical properties of creep behavior. Most of the published models are isotropic, and the homogeneity model cannot effectively describe the heterogeneous characteristics of sandstone in the mesoscale. In this work, an instantaneous elastic-plastic damage constitutive model and a viscoelastic plastic time-dependent damage constitutive model of sandstone are established, based on laboratory test data. These models are validated, respectively, by an analytical solution of an elastic-plastic model and by the Nishihara model. Supplementing this work, a series of conventional constant-strain-rate tests and multi-step creep tests were carried out on sandstone, which is very common in coal mine roofs. Then, the simulation results are carefully analyzed in combination with results from the laboratory. This research builds a damage constitutive model that should be able to describe the full stages of creep behavior for sandstone. The damage model can also express crack propagation or damage evolution before creep failure. 

## 2. Numerical Model

According to the instantaneous deformation of rock or rock-like material from constant-strain-rate tests [13] and the strain separation of rock creep behavior [14], an intact constitutive model of rock should contain two important parts: an instantaneous (time-independent) elastic-plastic part, and a time-dependent viscoelastic plastic part. The instantaneous elastic-plastic model could only be used for simulated constant-strain-rate tests, while the instantaneous elastic-plastic model and time-dependent viscoelastic plastic model combined could perform the whole behavior of rock during creep tests.

### 2.1. Instantaneous Elastic-Plastic Model

In order to describe crack evolution before failure, a damage model is adopted in this paper. Some elastic-plastic equations are added to the damage model to illustrate the instantaneous behavior of rock.

#### 2.1.1. Heterogeneous Damage Model

**Figure 1:**
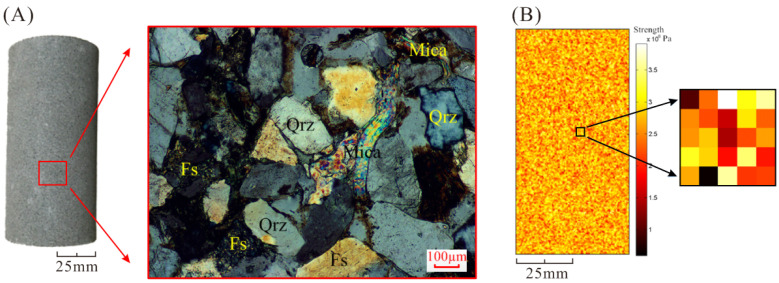
Heterogeneous model of sandstone in the mesoscale. (**A**) Optical microscope image at a 100 μm scale [45]. (**B**) Heterogeneous distribution of strength elements in the model.

Sandstone samples are homogeneous in the macroscale, but also composed of different kinds of mineral grains in the mesoscale (as shown in Figure 1A). All samples were cored, as the ISRM suggested method, from the same block of material to a diameter of 25 mm, and a length of 100 mm, resulting in a length to diameter ratio of 2:1. Therefore, the sandstone specimens are heterogeneous in the mesoscale. Accordingly, it is assumed that the numerical model of rock is composed of mesoscopic heterogeneous units so as to simulate the mesoscopic heterogeneity of the rock specimens [38,42,43]. As shown in Figure 1B, in order to illustrate the heterogeneity of rock elements in the mesoscale, the mechanical properties (such as strength and Young’s modulus) are assigned randomly by Weibull statistical distribution [44].

(1) $$f\left(u\right)=\frac{x}{u_{0}}{\left(\frac{u}{u_{0}}\right)}^{x-1}\exp\left[-{\left(\frac{u}{u_{0}}\right)}^{x}\right]$$

where u is a mechanical property of the rock in the mesoscale (strength, elastic modulus, etc.); $u_{0}$ is the average value of the mechanical properties for all elements; x is the uniformity coefficient, reflecting the homogeneity of the whole specimen; and f(u) is the statistical distribution density function of rock elements for the rock’s mechanical property *u*. More information on rock heterogeneity models can be found in these articles [37,38].

The maximum tensile stress criterion and the strength criterion determined by the plastic yield function [13] are adopted as damage judgment criteria. The maximum tensile stress criterion is:

(2) $$F_{1}\equiv \sigma_{1}-f_{t0}=0$$

where $\sigma_{1}$ is the minimum principal stress and $f_{t0}$ is the uniaxial tensile strength. Under any stress condition, the tensile damage is judged first by the maximum tensile stress criterion. When the strength does not meet the tensile criterion, it must be decided whether the strength meet the strength criterion. The strength criterion used in the model is based on the plastic yield function, which is described in detail in the following section.

When the stress state or strain state of the rock element satisfies the given damage threshold, the element begins to be damaged. The elastic damage of the elastic modulus could be expressed by the following:

(3) $$E=E_{0}(1-D)$$

where $E_{0}$ and E are, respectively, the elastic moduli before and after the damage and *D* is the damage variable. However, the damage of the element and its evolution are all assumed to be isotropic in the model, so $E_{0}$, E and *D* are all scalars. The damage variable *D* of the mechanical parameters under compressive and tensile stress states satisfies the piecewise function damage constitutive relation, which can be written:

(4) $$D=\left\{ \begin{array}{cc} 0 & F_{1}<0\;\;\;\;and\;\;\;F_{2}<0\\ 1-{\left|\frac{\varepsilon_{t0}}{\varepsilon_{1}}\right|}^{n}-\frac{\sigma_{tr}}{E_{0}\varepsilon_{1}} & F_{1}=0\;\;\;\;and\;\;\;dF_{1}>0\\ 1-{\left|\frac{\varepsilon_{c0}}{\varepsilon_{3}}\right|}^{n}-\frac{\sigma_{cr}}{E_{0}\varepsilon_{3}}\;\;\;\;\;\;\;\;\;\; & F_{2}=0\;\;\;and\;\;\;dF_{2}>0 \end{array}\right.$$

where $\varepsilon_{1}$ and $\varepsilon_{3}$ are, respectively, the tensile and compressive principal strains; $\varepsilon_{t0}$ and $\varepsilon_{c0}$ are the corresponding maximum tensile and compressive principal strains when the element experiences tensile or shear damage; n is the element damage evolution coefficient, taken to have the value of 2; $\sigma_{t}{}_{r}$ and $\sigma_{c}{}_{r}$ are the residual stresses at the stress condition of tension and compression, respectively; $E_{0}$ is the initial elastic modulus of the element; $F_{2}$ is the strength criterion of the element, which is determined by the plastic yield function $F_{2}=f_{f}(\sigma_{ij},\sigma_{f})$; and $\sigma_{ij}$ is the principal stress. At the loading stage, as the plastic strain increases, the element can also start to be damaged when the strength criterion is satisfied.

The stress–strain relationship can be described by the elastic-plastic constitutive equation. The matrix expression of the equation is usually written in the following form:

(5) $$\left\{d\varepsilon \right\}=\left[C_{ep}\right]\left\{d\sigma \right\}$$

where $\left[C_{ep}\right]$ is called the elastic-plastic flexibility matrix, $\left[C_{ep}\right]=\left[C_{e}\right]+\left[C_{p}\right]$; $\left[C_{e}\right]$ is the flexibility matrix of elastic strain, while $\left[C_{p}\right]$ is the flexibility matrix of plastic strain. After the strength reaches the limit in the compressive stress state, and considering the strain softening (due to the damage after the strength meets the criterion), the whole stiffness matrix of the elastic-plastic strain can be written:

(6) $$\left\{d\varepsilon \right\}=\frac{\left[C_{ep}\right]}{\left(1-D\right)}\left\{d\sigma \right\}$$

#### 2.1.2. Instantaneous Elastic-Plastic Model

The deformation of rock can be decomposed as follows: instantaneous deformation (time-independent) and TDD [12,13,14].

(7) $$\varepsilon_{ij}=\varepsilon_{ij}^{i}+\varepsilon_{ij}^{c}$$

The instantaneous deformation part can be separated further into two parts: instantaneous elastic deformation and instantaneous plastic deformation:

(8) $$\varepsilon_{ij}^{i}=\varepsilon_{ij}^{e}+\varepsilon_{ij}^{p}$$

According to the generalized Hooke’s law, the three-dimensional constitutive relationship of the elastic body’s instantaneous deformation can be written:

(9) $$\begin{array}{cc} \varepsilon_{ij}^{e} & =\frac{1}{2G_{0}}s_{ij}+\frac{1}{3K_{0}}\sigma_{m}\delta_{ij}\\ & =\frac{1}{E}\left[\left(1+\mu \right)s_{ij}+\left(1-2\mu \right)\sigma_{m}\delta_{ij}\right]\\ & =\left[C_{e}\right]\sigma_{ij} \end{array}$$

where $s_{ij}$ is the stress deviatoric tensor; $\sigma_{m}$ is the spherical stress tensor; $\delta_{ij}$ is the Kronecker delta (zero when *i* ≠ *j* and 1 when *i* = *j*); $\left[C_{e}\right]$ is the elastic flexibility matrix of the element; $G_{0}$ and $K_{0}$ are, respectively, the shear modulus and the bulk modulus; and μ is Poisson’s ratio.

According to the flow law of the traditional elastic-plastic theory, the plastic strain increment can be found [16,46] as follows:

(10) $$d\varepsilon_{ij}^{p}=d\lambda_{p}\frac{\partial Q_{p}}{\partial \sigma_{ij}}$$

where $Q_{p}$ is the plastic potential function of rock; $d\lambda_{p}$ is the plastic strain increment. The plastic strain increment index can be derived from the plastic consistency condition.

(11) $$d\lambda_{p}=\frac{\left(\partial f_{p}/\partial \sigma_{ij}\right)d\sigma_{ij}}{-\left(\partial f_{p}/\partial \alpha_{p}\right)\left(\partial \alpha_{p}/\partial \varepsilon_{ij}^{p}\right)}$$

where $f_{p}$ is the plastic subsequent yield function and $\alpha_{p}$ is the plastic hardening function.

According to Equations (8)–(10), the expression of total elastic-plastic strain can be found to be:

(12) $$\left\{d\varepsilon_{ij}^{i}\right\}=\left[C_{e}\right]\left\{d\sigma_{ij}^{e}\right\}+\left[C_{p}\right]\left\{d\sigma_{ij}^{p}\right\}$$

Zhou et al. [13], Shao et al. [12], Jia et al. [47] and Pietruszczak et al. [48] considered that the following yield function can be used to represent the plastic yield surface of the elastic-plastic model, which can effectively characterize the instantaneous ductile deformation characteristics of rock:

(13) $$f_{p}\left(\sigma_{ij},\alpha_{p}\right)=q-\alpha_{p}\left(\gamma_{p}\right)R_{c}\sqrt{A\left(C_{s}+\frac{p}{R_{c}}\right)}=0$$

(14) $$\begin{array}{l} p=-\frac{\sigma_{m}}{3},q=\sqrt{\frac{3}{2}s_{ij}s_{ij}},\\ s_{ij}=\sigma_{ij}-\frac{\sigma_{m}}{3}\delta_{ij} \end{array}$$

where *p*, *q* and *θ* are the mean stress (the compression force is taken as negative in the model), stress deviator and Lode angle, respectively; $\alpha_{p}\left(\gamma_{p}\right)$ is the plastic hardening function; $R_{c}$ is the normalization coefficient, which is taken to be the strength of the element in this model; $C_{s}$ is the cohesive coefficient of the rock (with a value of 0.01); and A is a constant related to the internal friction of the rock.

The plastic hardening function in Equation (13) can be described by a function related to the effective plastic shear strain [47]:

(15) $$\alpha_{p}\left(\gamma_{p}\right)=1+\frac{\gamma_{p}}{B+\gamma_{p}}$$

where *B* is a coefficient controlling the plastic hardening rate and $\gamma_{p}$ is the effective plastic shear strain, which can be illustrated by the following equation [49]:

(16) $$d\gamma_{ij}^{p}=\sqrt{\frac{2}{3}de_{ij}^{p}de_{ij}^{p}}$$

in which,

(17) $$e_{ij}^{p}=\varepsilon_{ij}^{p}-\frac{\varepsilon_{kk}}{3}\delta_{ij}$$

In order to describe the transition of volumetric strain from compression to dilatancy, a non-associated plastic flow rule [12,13,50,51] is needed. Additionally, a plastic potential function not equal to the yield function should be proposed to achieve the non-associated plastic flow rule. Based on experimental data, the plastic potential function can be found to be:

(18) $$Q_{p}=q-\left[\alpha_{p}\left(\gamma_{p}\right)-\beta_{p}\right]\left(p+C_{s}R_{c}\right)$$

where $\beta_{p}$ is a coefficient controlling the turning point of volume deformation from compaction to dilatancy; it is taken to be 1.1. When $\alpha_{p}\left(\gamma_{p}\right)$ < $\beta_{p}$, the volume deformation is compaction; when $\alpha_{p}\left(\gamma_{p}\right)$ > $\beta_{p}$, the deformation is dilatancy.

Substituting Equations (13) and (18) into Equation (11), the plastic strain increment can be found to be:

(19) $$d\lambda_{p}=\frac{\left[\sqrt{\frac{3}{2}}\frac{s_{ij}}{\sqrt{s_{ij}s_{ij}}}+\frac{\alpha_{p}\left(\gamma_{p}\right)A}{6{\left[A\left(C_{s}-\frac{\sigma_{kk}}{3}\frac{1}{R_{c}}\right)\right]}^{\frac{1}{2}}}\delta_{ij}\right]d\sigma_{ij}}{\left[R_{c}\sqrt{A\left(C_{s}+\frac{p}{R_{c}}\right)}\right]\left[\sqrt{\frac{2}{3}}\frac{B}{{\left(B+\gamma_{p}\right)}^{2}}\right]}$$

**Figure 2:**
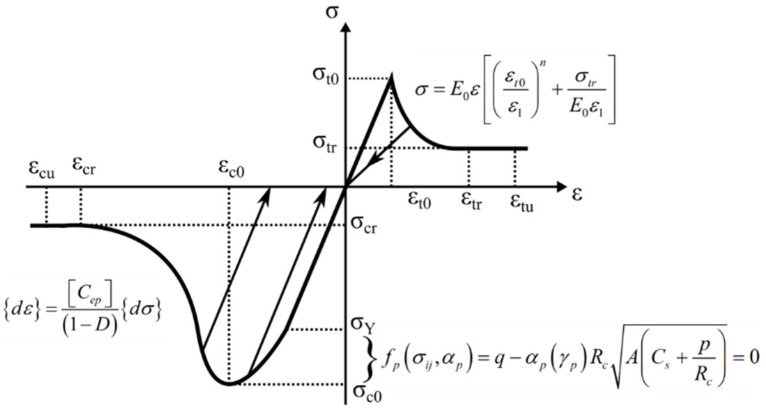
Elastic-plastic damage constitutive rule for an element under uniaxial compression and tension.

In general, the elastic-plastic constitutive damage model for the element can be described by Equation (12). The elastic-plastic constitutive model of the compression and tension loading for the element is shown in Figure 2.

The loading and unloading conditions of the elastic-plastic model are expressed as follows:

(20) $$f_{p}\left(\sigma_{ij},\alpha_{p}\right)=0,\;\;df=\left\{ \begin{array}{ll} \frac{\partial f_{p}\left(\sigma_{ij},\alpha_{p}\right)}{\partial \sigma_{ij}}d\sigma_{ij}<0, & \mathrm{Unloading}\;\mathrm{stage}\;\\ \frac{\partial f_{p}\left(\sigma_{ij},\alpha_{p}\right)}{\partial \sigma_{ij}}d\sigma_{ij}=0, & \mathrm{Changing}\;\mathrm{loading}\;\mathrm{stage}\\ \frac{\partial f_{p}\left(\sigma_{ij},\alpha_{p}\right)}{\partial \sigma_{ij}}d\sigma_{ij}>0, & \mathrm{Loading}\;\mathrm{stage} \end{array}\right.$$

#### 2.1.3. Model Validation

Some numerical results are compared with the result from analytical solution model to validate the availability of the proposed elastic-plastic model.

(1) Analytical solution model

In order to verify the reliability of the elastic-plastic numerical model established above, a circular hole was excavated (numerically) in the stress field. The stress field distribution around the circular hole obtained by the proposed numerical model was compared with that obtained by an analytical solution. The comparison was used to achieve the validation of the numerical model. 

**Figure 3:**
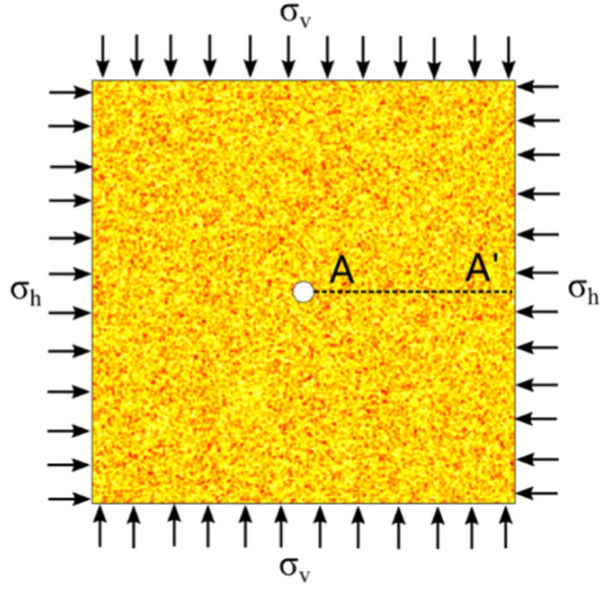
Numerical model of analytical solution with a borehole in the center of the stress field.

As shown in Figure 3, the size of the region to be investigated is 200 cm × 200 cm, with a borehole radius of 5 cm, and with the number of units set to 40,000. The horizontal stress is σ_h_, and the vertical stress is σ_v_. The dashed line A-A′ in Figure 3 extends from the right side of the central circular hole to the right boundary of the stress field. In addition, the tangential (vertical) and radial (horizontal) stress data of the elements on the dashed line are extracted from the numerically calculated result and from the analytical solution for further comparison.

The analytical solution of the elastic-plastic model with the M-C criterion, taking cohesion into account, can be described by:

(21) $$\sigma_{\theta }=\sigma_{r}\frac{1+\sin\phi }{1-\sin\phi }+2c\frac{\cos\phi }{1-\sin\phi }$$

where ϕ is the internal friction angle of the rock (assigned the value of 29°), $\sigma_{r}$ is the radial stress, $\sigma_{\theta }$ is the circumferential stress and c is the cohesion force, which is taken to be 0.12 MPa.

The analytical solution assumes that the stress distribution around the circular hole can be divided into a circumferential plastic zone and a circumferential elastic zone outside the plastic zone. The formula for stress distribution in the elastic zone is as follows:

(22) $$\left\{ \begin{array}{l} \sigma_{r}=\sigma_{0}-\left(\sigma_{0}-\sigma_{e}\right)\frac{r_{e}^{2}}{r^{2}}\\ \sigma_{\theta }=\sigma_{0}+\left(\sigma_{0}-\sigma_{e}\right)\frac{r_{e}^{2}}{r^{2}} \end{array}\right.$$

where $\sigma_{e}$ is the radial stress of the elastic-plastic boundary, $\sigma_{e}=\left(1-\sin\phi \right)\sigma_{0}$ and $\sigma_{0}$ is the far-field stress.

The stress distribution in the plastic zone can be expressed in the following form:

(23) $$\{\begin{array}{l} \sigma_{r}=\left(p+c\cot\phi \right){\left(\frac{r}{r_{0}}\right)}^{\frac{2\sin\phi }{1-\sin\phi }}-c\cot\phi \\ \sigma_{\theta }=\frac{1+\sin\phi }{1-\sin\phi }\left(p+c\cot\phi \right){\left(\frac{r}{r_{0}}\right)}^{\frac{2\sin\phi }{1-\sin\phi }}-c\cot\phi \end{array}$$

where *p* is the inner wall pressure of the borehole, with a value of 2 MPa, and $r_{0}$ is the radius of the circular hole, taken to be 5 cm.

According to the stress continuity condition of the elastic-plastic boundary, the radius of the boundary between the elastic and plastic regions can be written:

(24) $$r_{e}=r_{0}{\left[\frac{\left(1-\sin\phi \right)\sigma_{0}-c\left(\cos\phi -\cot\phi \right)}{\left(p+c\cot\phi \right)}\right]}^{\frac{1-\sin\phi }{2\sin\phi }}$$

where $\sigma_{0}$ is the far-field stress, with a value of 10 MPa.

(2) Numerical result validation

**Figure 4:**
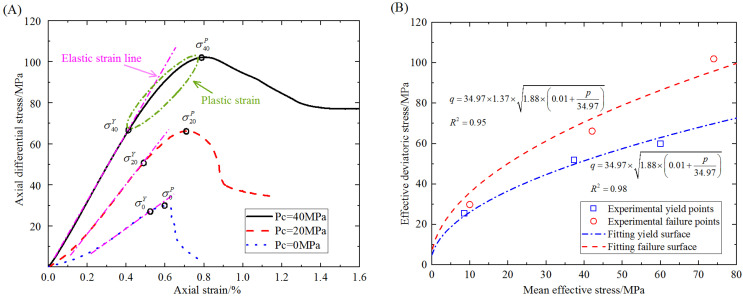
Fitted yield and fracture surfaces from experimental data. (**A**) Axial differential stress vs. axial strain curves under different confining pressures. (**B**) Effective deviatoric stress vs. mean effective stress for the yield surface and the failure surface.

Conventional triaxial compressive strength tests were conducted on the sandstone to obtain the stress–strain curves for confining pressures of 0, 20, and 40 MPa. The stress–strain curves are shown in Figure 4A. Based on these curves and the definitions of effective deviatoric stress and mean effective stress, the yield stress surface and the failure stress surface for the sandstone are illustrated in Figure 4B. The plastic yield function of Equation (13) was used to fit the data in Figure 4B, which reveals the undetermined parameters in the plastic yield function. The determined parameters in the plastic yield function for the sandstone are listed in Table 1. They can determine the initial yield function and the corresponding strength criterion for the sandstone. After some calculations by repetition, the physical and mechanical parameters in the numerical model are also obtained as shown in Table 1.

**Figure 5:**
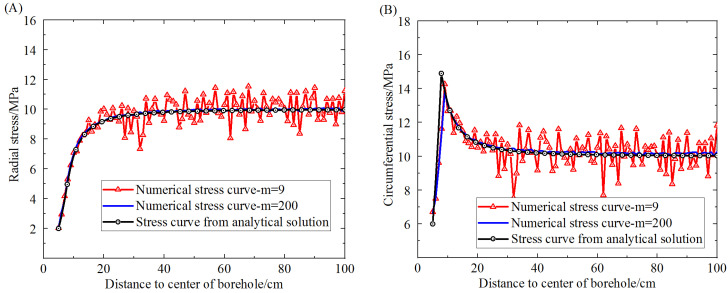
Comparison between numerical simulation results at different degrees of homogeneity and the analytical solutions. (**A**) Stress curves of radial stress vs. distance to the center of the borehole. (**B**) Stress curves of circumferential stress vs. distance to the center of the borehole.

The proposed elastic-plastic damage model, which adopts the boundary condition of a stress field with a circular borehole, can be used to get the stress distribution on the dashed line A-A′ in Figure 3. The stress distribution around the circular borehole obtained from the proposed damage model and the analytical solution are shown in Figure 5. The stresses for comparison contain radial stress and circumferential stress. The results obtained from the proposed damage model show two different degrees of homogeneity. When the homogeneity of the rock sample is very high, the strengths of elements on the dashed line tend toward the same value. However, the lower the homogeneity of the rock, the more fluctuant the stress on elements around the result of the analytical solution. The fluctuation of the stress distribution is due to the Weibull distribution of the element strength in the model, which can induce a difference of stress distribution on the elements. 

**Figure 6:**
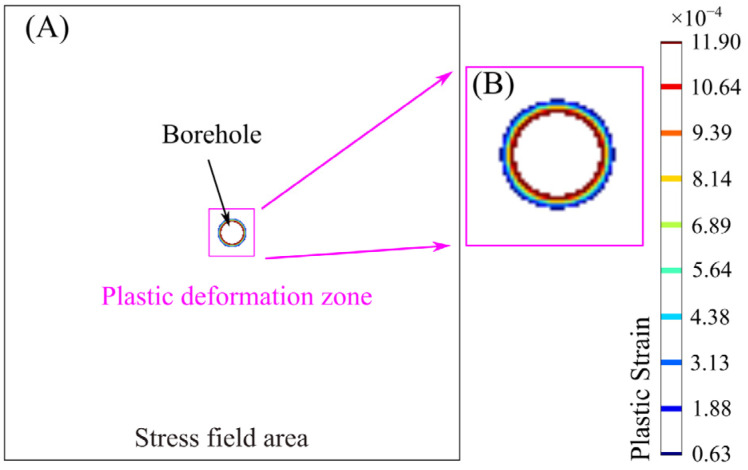
Cloud map of plastic strain around the borehole calculated from the damage model. (**A**) Plastic deformation zone around the borehole. (**B**) Enlarged view.

As shown in Figure 5A, the comparative results of radial stress show that when the homogeneity is 200, the radial stress obtained from the elastic-plastic damage model is in good agreement with that from the analytical solution. When the homogeneity is 9, the radial stress obtained from the damage model fluctuates around that of the analytical solution. This indicates that the proposed damage model is promising for getting the distribution of radial stress. As shown in Figure 5B, the comparative results show that the circumferential stress extracted from the result of the proposed elastic-plastic damage model differs little from that of the analytical solution when the homogeneity is very high. The difference is the location of the maximum yield stress around the borehole, a difference that comes about because the yield function used in the proposed model is different from that in the analytical solution. Yet the various features of the circumferential stress curves obtained from the two models are almost the same, showing that the proposed damage model can account for the elastic behavior and the plastic behavior as well as the analytical solution. Additionally, the plastic deformation is extracted from the whole deformation occurring around the borehole and drawn in Figure 6. 

When the far-field stress is 10 MPa and the inner pressure in the borehole is 2 MPa, the results based on the damage model show that the radius of the plastic zone centered on the borehole is about 9 cm. At the same time, the radius of the plastic zone obtained from the analytical solution is 7.91 cm, very closed to that from the damage model. To sum up, the elastic damage model and elastic-plastic damage model with heterogeneity established in the present research are in good agreement with the stress field distribution obtained from the analytical solutions, which supports the reliability of the numerical damage model.

### 2.2. Time-Dependent Viscoelastic Plastic Model

The proposed viscoelastic plastic model is adapted from the traditional Nishihara model. The proposed model and the validation with an analytical solution are detailed as the follows.

#### 2.2.1. Nishihara Model

**Figure 7:**
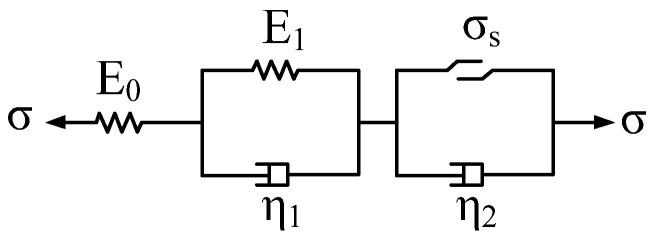
Schematic diagram of the traditional Nishihara model.

The traditional Nishihara model is composed of three groups of elements, such as elastic elements, viscoelastic elements and visco-plastic elements (as shown in Figure 7). The one-dimensional creep equation of the Nishihara model is as follows:

(25) $$\varepsilon \left(t\right)=\left\{ \begin{array}{ll} \frac{\sigma }{E_{0}}+\frac{\sigma }{E_{1}}\left[1-\exp\left(-\frac{E_{1}}{\eta_{1}}t\right)\right] & \left(\sigma <\sigma_{s}\right)\\ \frac{\sigma }{E_{0}}+\frac{\sigma }{E_{1}}\left[1-\exp\left(-\frac{E_{1}}{\eta_{1}}t\right)\right]+\frac{\sigma -\sigma_{s}}{\eta_{2}}t\;\;\; & \left(\sigma \geq \sigma_{s}\right) \end{array}\right.$$

where $\sigma_{s}$ is the yield stress at a one-dimensional scale. When $\sigma \geq \sigma_{s}$, the total strain can be regarded as $\varepsilon_{ij}=\varepsilon^{e}+\varepsilon^{ve}+\varepsilon^{vp}$. Furthermore, according to the three-dimensional form of the generalized Hooke’s law, the elastic deformation can be expressed as follows:

(26) $$\begin{array}{cc} \varepsilon_{ij}^{e} & =\frac{1}{2G_{0}}s_{ij}+\frac{1}{3K_{0}}\sigma_{m}\delta_{ij}\\ & =\frac{1}{E}\left[\left(1+\mu \right)s_{ij}+\left(1-2\mu \right)\sigma_{m}\delta_{ij}\right] \end{array}$$

where $G_{0}$ and $K_{0}$ are shear modulus and bulk modulus, respectively.

Viscoelastic and visco-plastic deformations can be described by the equations:

(27) $$\varepsilon^{ve}=\frac{\sigma }{E_{1}}\left[1-\exp\left(-\frac{E_{1}}{\eta_{1}}t\right)\right]$$

(28) $$\varepsilon^{vp}=\frac{\sigma -\sigma_{s}}{\eta_{2}}t$$

where $\eta_{1}$ and $\eta_{2}$ are, respectively, the shear viscosity coefficient and the plastic viscosity coefficient.

During transformation of the viscoelastic formula from one dimension to three dimensions, it is assumed that the rock does not exhibit creep behavior under a spherical stress tensor or hydrostatic pressure. It is further assumed that only stress deviation can cause the rock to exhibit creep behavior [13]. The viscoelastic strain in the TDD can be illustrated as follows:

(29) $$\varepsilon_{ij}^{ve}=\frac{s_{ij}}{2G_{1}}\left[1-\exp\left(-\frac{G_{1}}{\eta_{1}}t\right)\right]$$

where $G_{1}$ is the viscoelastic shear modulus, expressed by $G_{1}=\frac{E_{1}}{2\left(1+\mu \right)}$, in which μ is Poisson’s ratio.

According to the principles of plastic mechanics, the expression of visco-plastic strain in TDD can be expressed as follows [13,52]:

(30) $$\varepsilon_{ij}^{vp}=\frac{1}{\eta_{2}}\langle \phi \left(\frac{F}{F_{0}}\right)\rangle \frac{\partial Q}{\partial \sigma_{ij}}t$$

where F is the plastic yield function, $F_{0}$ is the initial value of the plastic yield function (which is taken to be 1) and Q is the plastic potential function related to the plastic yield surface. An expression of the Heaviside step function <∙> in Equation (30) is shown:

(31) $$\langle \phi \left(\frac{F}{F_{0}}\right)\rangle =\left\{ \begin{array}{ll} 0 & \left(F<0\right)\\ \phi \left(\frac{F}{F_{0}}\right)\;\;\;\;\;\;\;\; & \left(F\geq 0\right) \end{array}\right.$$

in which ϕ(•) is assumed to have the form of a power function. Usually the exponential index n in the power function is equal to 1 [52]. The power function can be written $\phi \left(\frac{F}{F_{0}}\right)=\frac{F}{F_{0}}$.

The total deformation equation for the three-dimensional form of the Nishihara model can be written as follows:

(32) $$\varepsilon_{ij}=\left\{ \begin{array}{l} \varepsilon_{ij}^{e}+\varepsilon_{ij}^{ve}\;\;\;\;\;\;\;\;\;\;\left(F<0\right)\;\\ \varepsilon_{ij}^{e}+\varepsilon_{ij}^{ve}+\varepsilon_{ij}^{vp}\;\;\;\left(F\geq 0\right)\;\; \end{array}\right.$$

By substituting the above formulas into Equation (32), the total deformation equation of the traditional Nishihara model in three-dimensional form can be found to be as follows:

(33) $$\varepsilon_{ij}=\left\{ \begin{array}{ll} \frac{1}{E_{0}}\left[\left(1+\mu \right)s_{ij}+\left(1-2\mu \right)\sigma_{m}\delta_{ij}\right]+\frac{s_{ij}}{2G_{1}}\left[1-\exp\left(-\frac{G_{2}}{\eta_{1}}t\right)\right]\;\;\;\;\;\;\;\;\; & ,\;\;\left(F<0\right)\\ \frac{1}{E_{0}}\left[\left(1+\mu \right)s_{ij}+\left(1-2\mu \right)\sigma_{m}\delta_{ij}\right]+\frac{s_{ij}}{2G_{1}}\left[1-\exp\left(-\frac{G_{1}}{\eta_{1}}t\right)\right]+ & \\ \frac{1}{\eta_{2}}\langle \phi \left(\frac{F}{F_{0}}\right)\rangle \frac{\partial Q}{\partial \sigma_{ij}}t & ,\;\;\left(F\geq 0\right) \end{array}\right.$$

#### 2.2.2. Improved Nishihara Model

As stated in the Nishihara model, the TDD can be divided into two partitions: viscoelastic deformation and visco-plastic deformation. The three-dimensional form of TDD can be expressed as

(34) $$\varepsilon_{ij}^{c}=\varepsilon_{ij}^{ve}+\varepsilon_{ij}^{vp}$$

A three-dimensional expression for the viscoelastic deformation is provided by Equation (29), and an expression of the visco-plastic deformation is provided by Equation (30). Therefore, the yield function and plastic potential function used in Section 2.1.2 can also be used here to establish the non-associated flow rule under visco-plastic strain in the TDD:

(35) $$f_{vp}\left(\sigma_{ij},\alpha_{vp}\right)=q-\alpha_{p}\left(\gamma_{p}\right)R_{c}\sqrt{A\left(C_{s}+\frac{p}{R_{c}}\right)}$$

(36) $$Q_{vp}=q-\left[\alpha_{p}\left(\gamma_{p}\right)-\beta_{p}\right]\left(p+C_{s}R_{c}\right)$$

The effective shear strain in Equation (16) can be described as follows:

(37) $$d\gamma_{\mathrm{ij}}^{p}=\sqrt{\frac{2}{3}de_{ij}^{p}de_{ij}^{p}}+\sqrt{\frac{2}{3}de_{ij}^{vp}de_{ij}^{vp}}$$

Based on Equations (29), (30), (35) and (36), an expression of the TDD can be derived:

(38) $$\begin{array}{l} \varepsilon_{ij}^{c}=\varepsilon_{ij}^{ve}+\varepsilon_{ij}^{vp}\\ =\frac{s_{ij}}{2G_{1}}\left[1-\exp\left(-\frac{G_{1}}{\eta_{1}}t\right)\right]+\frac{1}{\eta_{2}}f_{vp}\left(\sigma_{ij},\alpha_{vp}\right)\left[\sqrt{\frac{3}{2}}\frac{s_{ij}}{\sqrt{s_{ij}s_{ij}}}+\frac{1}{3}\left(1+\frac{\gamma_{p}}{B+\gamma_{p}}-\beta_{p}\right)\delta_{ij}\right]t \end{array}$$

**Figure 8:**
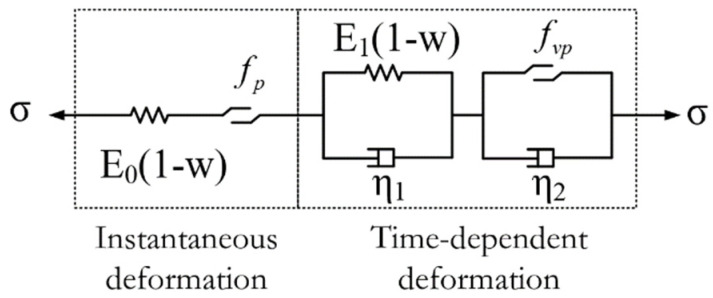
Schematic diagram of the improved Nishihara model.

After synthesizing instantaneous deformation and TDD, the total viscoelastic plastic deformation equation can be found from Equation (7) (as shown in Figure 8). The three-dimensional expression of the total deformation equation can then be rewritten:

(39) $$\left\{d\varepsilon_{\mathrm{ij}}\right\}=\frac{\left[C_{ep}\right]}{\left(1-D\right)}\left\{d\sigma_{ij}^{ci}\right\}+\left\{d\varepsilon_{ij}^{c}(D,\sigma_{ij}^{c})\right\}$$

where $\varepsilon_{ij}^{c}(D,\sigma_{ij}^{c})$ is expressed as the TDD, which depends on the damage factor and the loading stress.

#### 2.2.3. Analytical Solution Verification

(1) Analytical solution of Nishihara model based on D–P criterion

**Figure 9:**
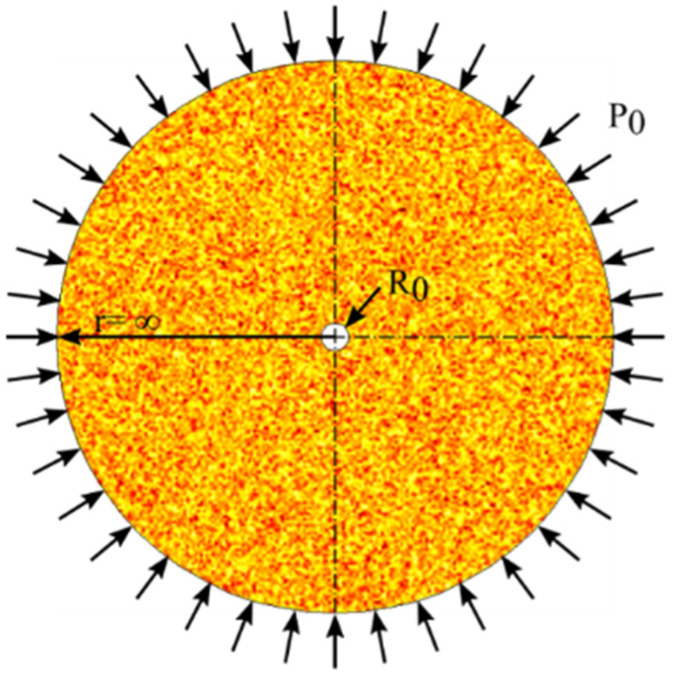
Mechanical model of circular stress field with a center borehole.

According to the circular tunnel of a perfect elastoplastic solution with the Drucker–Prager (D–P) yield criterion [53], the mechanical model shown in Figure 9 is established. The stress in the stress field around the circular hole with a radius of *R*_0_ is *P*_0_, and the distance from a point in the stress field to the center of the circular hole is *r*. According to the work of Hou and Niu [53], the plastic stress distribution around the circular hole, as derived using the D–P criterion, is as follows:

(40) $$\left\{ \begin{array}{l} \sigma_{r}^{p}=\frac{k}{3\alpha }\left[{\left(\frac{r}{R_{0}}\right)}^{\frac{6\alpha }{1-3\alpha }}-1\right]\\ \sigma_{\theta }^{p}=\frac{k}{3\alpha }\left[\frac{1+3\alpha }{1-3\alpha }{\left(\frac{r}{R_{0}}\right)}^{\frac{6\alpha }{1-3\alpha }}-\right] \end{array}\right.$$

where α and k are test constants related only to the internal friction angle φ and the cohesion factor C, and whose values can be taken to be:

(41) $$\alpha =\frac{2\sin\varphi }{\sqrt{3}\left(3-\sin\varphi \right)};k=\frac{6C\cos\varphi }{\sqrt{3}\left(3-\sin\varphi \right)}$$

The radius of the plastic zone can be derived from the following equation.

(42) $$R_{p}=R_{0}{\left[\left(1-3\alpha \right)\left(1+\frac{3\alpha }{k}P_{0}\right)\right]}^{\frac{1-3\alpha }{6\alpha }}$$

The stress distribution in the elastic zone can be described as follows:

(43) $$\begin{array}{l} \sigma_{\theta }^{e}\\ \sigma_{r}^{e} \end{array}\}=P_{0}\pm \frac{R_{0}^{2}}{r^{2}}k{\left(1-3\alpha \right)}^{\frac{1-3\alpha }{3\alpha }}{\left(1+\frac{3\alpha }{k}P_{0}\right)}^{\frac{1}{3\alpha }}$$

Up to this point, the stress distribution around the circular hole based on the D–P criterion can be achieved [53]. The analytical solution of the TDD around the circular hole based on the Nishihara model was deduced in another paper [54]. The deformation u and the TDD $u_{0}$ at *r* = *R*_0_ are, respectively, the following:

(44) $$u=\frac{\sqrt{3}R_{0}^{2}}{2r}A\varepsilon_{i}\left(t\right)$$

(45) $$u_{0}=\frac{\sqrt{3}R_{0}}{2}A\varepsilon_{i}\left(t\right)$$

(46) $$A={\left[\frac{3\alpha }{k}\left(1-3\alpha \right)\left(\frac{k}{3\alpha }+P_{0}\right)\right]}^{\frac{1-3\alpha }{3\alpha }}$$

(47) $$\varepsilon_{i}\left(t\right)=\sigma_{i}\left[\frac{1}{E_{0}}+\frac{1}{E_{1}}\left(1-e^{-\frac{E_{2}}{\eta_{1}}t}\right)\right]+\frac{\langle \sigma_{i}-\sigma_{s}\rangle }{\eta_{2}}t$$

According to published data [53,54], some basic mechanical parameters in the numerical model used for model validation can be determined and are shown in Table 2. Based on the analytical solution, the stress distribution and the TDD around the circular hole can be obtained.

(2) Verification results

**Figure 10:**
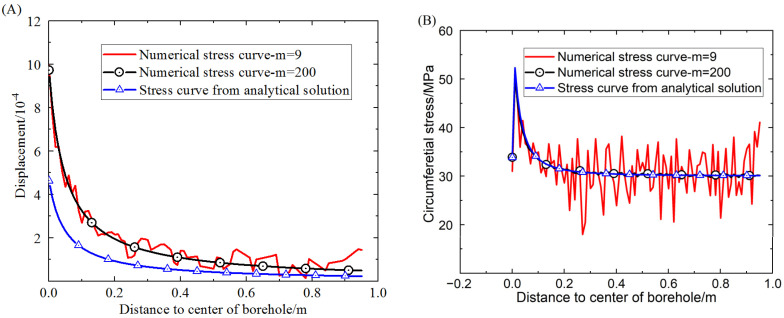
Comparison of displacement and circumferential stress distribution between the analytical solution and the proposed damage model at different homogeneities.

Using the analytical solution of the Nishihara model based on the D–P criterion and the proposed damage model, the relationship between the displacement of elements around the borehole and the distance *R*_0_ to the boundary of the borehole is displayed in Figure 10A. Similarly, the stress distribution around the circular hole plotted against the distance to the boundary of the borehole is shown in Figure 10B. 

The comparison of displacements shows that when the homogeneity of the element mechanical properties is up to 200, there is a little difference between the displacement obtained by the damage model and that by the analytical solution. The small difference is caused by the difference in yield functions used in the model and the analytical solution. In fact, the stress distribution law extracted from the result of the damage model is in good agreement with that from the analytical solution. Especially when the homogeneity is 200, the stress distribution for the damage model is almost the same as that for the analytical solution. Therefore, the results obtained from the proposed damage model match well with those from the analytical solution.

## 3. Comparisons with Laboratory Tests

In order to illustrate advantages and disadvantages of the proposed damage model, we have conducted some uniaxial compressive tests on the sandstone. Based on a comparison of stress–strain curves and the failure mode from the numerical model and from the laboratory tests, it will be helpful for readers to understand the proposed model for its possible use in the future.

### 3.1. Consistent Strain Rate Tests

**Figure 11:**
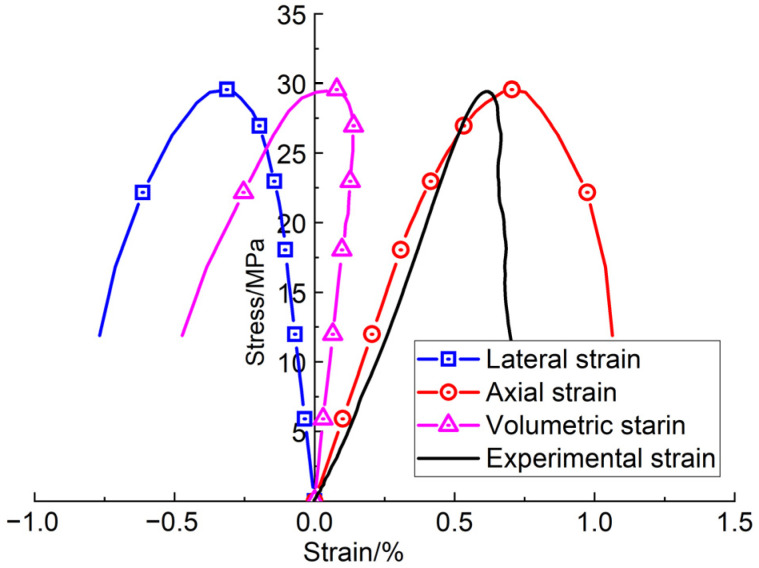
Stress–strain curves of Neijiang sandstone obtained from numerical simulation and laboratory tests. Lateral strain, axial strain and volumetric strain are obtained from the numerical simulation.

Based on the parameters in Table 1 and the viscoelastic plastic damage model, some uniaxial compressive strength (UCS) tests (or constant-strain-rate tests) were carried out. During the laboratorial tests, specimens were deformed uniaxially at a constant strain rate of 10^−5^ s^−1^ until macroscopic failure. The stress–strain curves obtained from the numerical simulation and the laboratory tests are compared in Figure 11. From the comparison of the stress–strain curves, it can be found that the numerical damage model can exhibit the full stress–strain process of the sandstone, and can also show compression and dilation behavior of volumetric deformation. This feature is basically consistent with that illustrated by the Vosges sandstone in ref. [13]. Additionally, the compression and dilation behavior of the volumetric strain in the numerical simulation can verify the validity of the non-associated flow rule adopted in the damage model.

**Figure 12:**
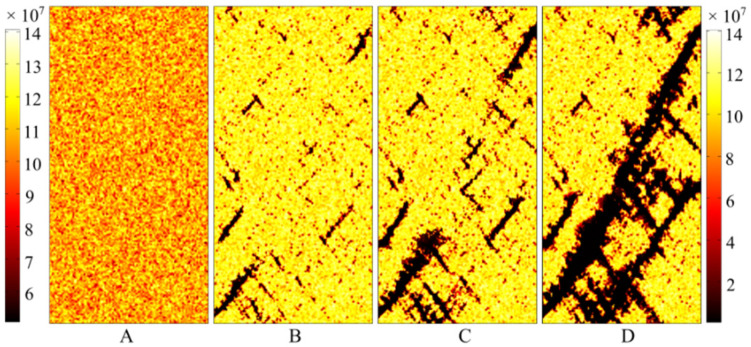
Strength failure snapshots extracted from the simulation result of the damage model. (**A**) An undamaged model. (**B**) Some cracks appear in the specimen. (**C**) Some cracks appear to accumulate and coalesce. (**D**) Main fracture surface is generated and some new cracks appear around the main fracture.

Some snapshots of the element strength during failure of the numerical model are extracted and shown in Figure 12. All of these snapshots have the same representative characteristic. As shown in the figure, the distribution of cracks is relatively discrete when the cracks begin to appear in the numerical specimen (Figure 12B). Then, the cracks begin to aggregate when they are very closed to each other (Figure 12C). At the same time, outlines of the final fracture have begun to form. However, as shown in Figure 12D, some small cracks also appear around the main fracture surface, after the main fracture surface has come into being. 

**Figure 13:**
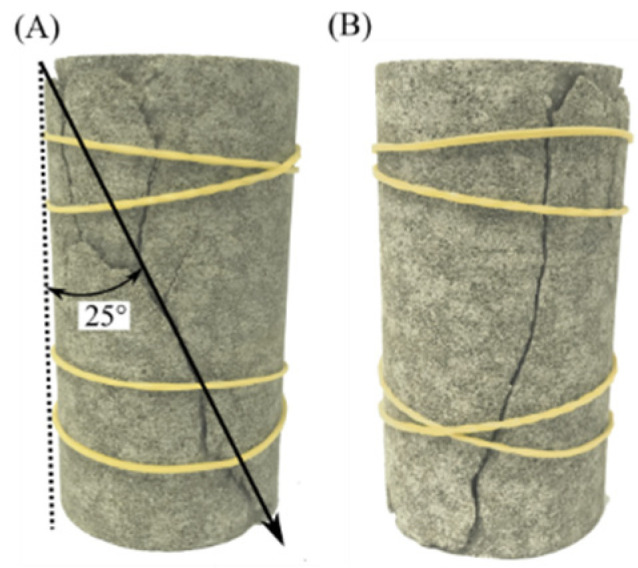
Failure mode of Neijiang sandstone in uniaxial compression strength tests. (**A**) The shear failure angle is about 25 degrees. There are some small cracks around the main fracture. (**B**) A main shear fracture on another side opposite to (**A**).

Figure 13 shows the failure picture of sandstone in the laboratory tests after the UCS tests, demonstrating that the failure mode obtained from the damage model is very similar to that from the laboratory tests. All of the failure modes from the damage model and the laboratory tests are oblique shear failure, and their inclined angles are almost the same. The damage model can also confirm the phenomenon that some small cracks appear around the main fracture. All of this evidence demonstrates that the proposed damage model has a very good ability to reproduce the instantaneous failure of sandstone, not only as demonstrated in the stress–strain curves, but also as illustrated by the failure mode and fracture evolution. 

### 3.2. Multi-Step Creep Tests

**Figure 14:**
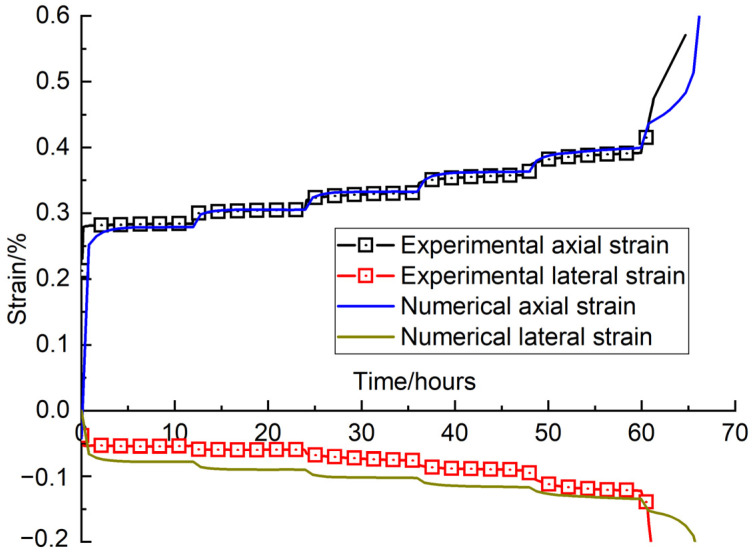
Comparison of axial and transverse strain vs. time curves in numerical simulation and in laboratory tests.

**Figure 15:**
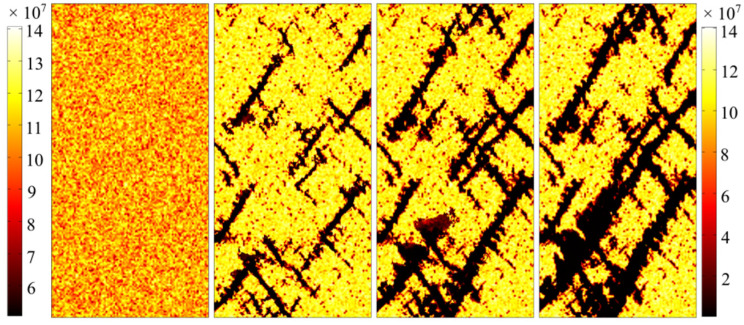
Contour diagram of strength failure of graded incremental loading creep test.

**Figure 16:**
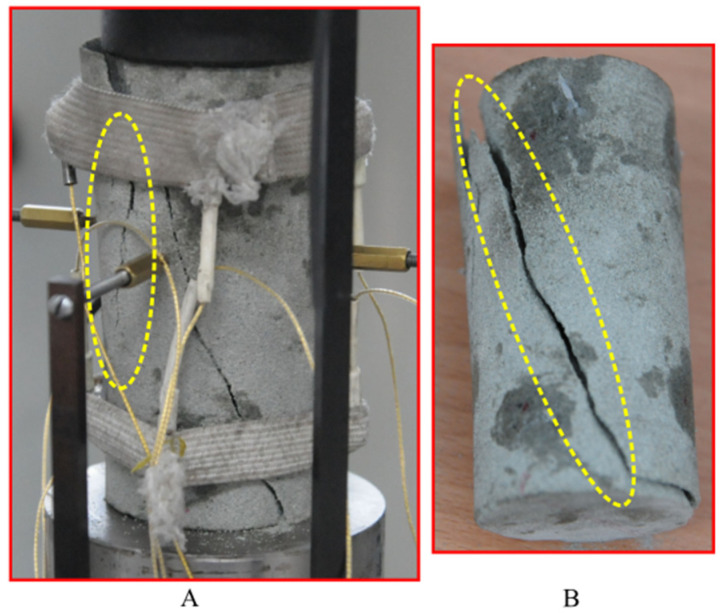
Failure mode of Neijiang sandstone in multi-step creep test. (**A**) The failure mode focuses on the shear failure, and a small crack (highlighted by the yellow circle) occurs near the main fracture similar to that in the failure mode in the UCS tests. (**B**) A main shear fracture on another side opposite to (**A**).

Considering the parameters in Table 1 and Table 2, some multi-step creep tests were conducted on sandstone using the proposed damage model. The loading stress levels of the creep test were set at 60%, 65%, 70%, 75%, 80% and 85% of the peak strength for the sandstone. The loading stress at every stage was held for 12 h. Then the test was switched to the next loading stage until the sandstone specimen failed. Figure 14 compares the multi-step creep curves obtained from the damage model and from the laboratory tests. The results show that the creep curves obtained from the numerical model, especially for the axial strain, fit well with those from the laboratory tests, which also supports the feasibility of using the model used in the creep test. The failure evolution drawn from the results of the numerical multi-step creep tests is shown in Figure 15. The snapshots of the failure evolution show that numerous cracks appear in the specimen in the multi-step creep tests. In addition, more cracks accumulate and develop next to the small fracture, more than in the numerical UCS test. Especially for the final failure mode, the number of main fractures occurring during the multi-step creep test was more than that during the UCS test, which can be certified by the result of creep tests in the lab, as shown in Figure 16. A small fracture appears around the main fracture during the experimental creep tests. Numerous fragments of sandstone come out at the fracture surface during these tests. By contrast, the fracture surface during the UCS tests does not have so much rock fragmentation, which means that the damage on the fracture surface is less pronounced for this way of loading. Both the fractures in the failure mode during the numerical simulation and the fragments on the fracture surface during the experimental tests can be interpreted to mean that the TDD has an accelerating effect on the generation and expansion of cracks inside the specimen, and also has some influence on the final fracture form of the specimen.

**Figure 17:**
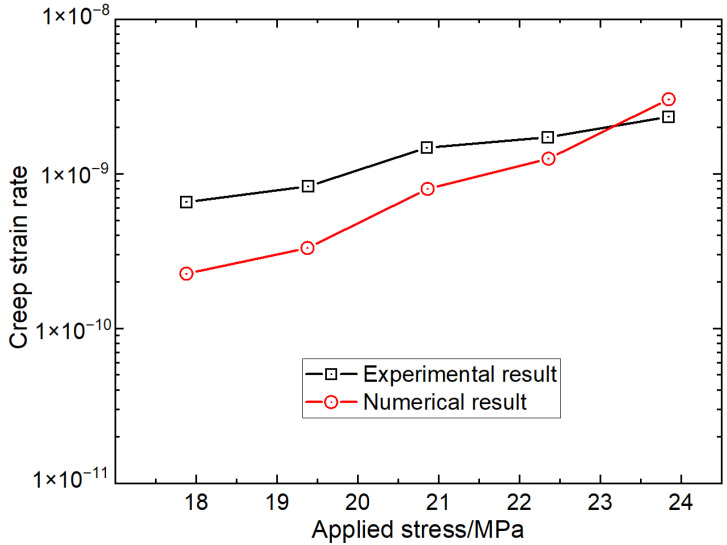
Relationships between creep strain rate and applied stress in the multi-step creep tests in the laboratory and in numerical simulation.

Based on the creep strain in Figure 14, creep strain rates at different loading stress levels for the experimental test and the numerical test can be obtained and are shown in Figure 17. The comparison shows that the creep strain rates obtained from the numerical simulation results have a consistent trend that matches those from the laboratory tests. The creep strain rates gradually increase with an increase of loading stress. the creep strain rate has a strong linear relationship with the corresponding loading stress in a semi-logarithmic graph. 

### 3.3. Discussion

All of above comparisons show that the proposed damage model can reveal the basic deformation characteristics of the time-dependent creep behavior and can also be used to investigate damage or evolution of cracks and the final fracture patterns, not only for the instantaneous deformation but also the TDD. However, the deformation process for rock is very complicated due to it being anisotropic in the mesoscale, and due to the variability among different specimens. Even though we have adopted the Weibull distribution of strength parameters in the numerical model to describe the heterogeneity of the sandstone in the mesoscale, it is still very difficult to match the macro behavior of the sandstone sample. The proposed damage model in the present paper can reveal the crack evolution and the failure mode, which are very similar to the results of experimental tests. However, we could not get the numerical and laboratory results for the strain–time curves to match at 95% or more. The development of the TDD constitutive model remains a significant problem. Some researchers have proposed models to simulate the rock creep behavior and have found good fitting curves [14,16,19]. However, the good fitting focuses only on the strain–time curves. If we want to have good matching results for both the strain curves and the failure model, more research work will need to be done. 

We have adopted a complicated multinomial in the damage model, and there are some parameters to determine before the model can be used. Considering the differences among rock samples and the varieties of rock, it is very difficult to predict accurately what the creep behavior of another rock sample will be. Additionally, the calculated quantities using the damage model will increase because of the long multinomial, which will reduce the computation speed. The damage model limits the amount of calculations and the built model will not be very complicated. The damage model, if used to solve engineering problems in the future, will be challenging because of its high calculation requirement, but could very well be used to better understand the failure of rock samples. Before it can be used in practical engineering problems, the damage model will need some simplification.

## 4. Conclusions

In order to explore the TDD characteristics of sandstone, especially for the evolution of cracks before rock failure, a viscoelastic plastic damage model was established in this work. The elastoplastic behavior and the viscoelastic plastic behavior of the damage model were validated by analytical solutions. After the validation of the damage model, uniaxial compressive strength tests and multi-step creep tests were conducted on the sandstone to investigate the merits and the deficiencies of the model. The research results reveal the following:

(1) The instantaneous elastic-plastic damage model can display the process of volumetric strain for sandstone from compression to dilation. Many small fractures are formed within a very short period of time in the specimen before the main fracture emerges. The damage model captures the phenomenon that small damage of the specimen can lead to the generation of micro-cracks, the accumulation of which causes the specimen to fracture. The final failure mode provided by the numerical simulation is consistent with that extracted from laboratory test results.
(2) The viscoelastic plastic damage model can reproduce the viscoelastic plastic deformation behavior. However, unlike the result of the conventional constant-strain-rate test, cracks of the rock appear through all of the stress loading stages in the multi-step creep tests. The final macroscopic fracture is the result of gradual accumulation of the cracks, a conclusion that is similar to that of the UCS tests.

## Figures and Tables

**Table 1:** Physical-Mechanical Parameters in Numerical Models.

Mechanical Parameters	Value
Specimen size	50 mm × 100 mm
Element size	0.5 mm
Uniaxial compressive strength (UCS), *σ*/MPa	110
Young’s modulus, E/GPa	10.0
Poisson’s ratio, *μ*	0.3
Homogeneity index, m	9.0
Increment per step, dy/mm	10^−5^
Parameter related to internal friction, *A*	1.88
Plastic hardening function in the strength criterion, $\alpha_{f}$	1.37
Plastic hardening function in the field criterion, $\alpha_{0}$	1.0
Cohesive coefficient, *C_s_*	0.01
Coefficient controlling plastic hardening rate, *B*	4 × 10^−5^

**Table 2:** Mechanical parameters used for model validation.

Physical and Mechanical Parameters	Value
Hole radius *R*_0_/m	0.05
In situ stress *P*_0_/MPa	30
UCS of surrounding rock *σ*_0_/MPa	20
Internal friction angle *φ*/°	25
Cohesion *C*/MPa	6.0
Elastic modulus *E*_0_/MPa	6.0
Elastic modulus *E*_1_/MPa	11.76
Viscosity coefficient *η*_0_/GPa∙d	3.57
Viscosity coefficient *η*_1_/GPa∙d	32.0

## Data Availability

Not applicable.
